# Challenges in Healthcare Systems and Women's Caregiving Roles^1^

**DOI:** 10.3201/eid1011.040622_04

**Published:** 2004-11

**Authors:** Rosaly Correa-de-Araujo, Patricia Stone, Sean Clarke

**Affiliations:** * Agency for Healthcare Research and Quality, Rockville, Maryland, USA;; † Columbia University School of Nursing, New York, New York, USA;; ‡ University of Pennsylvania School of Nursing, Philadelphia, Pennsylvania, USA

**Keywords:** women, caregiving roles, conference summary

Improving patient safety is one of the challenges facing our healthcare system. A report in January 2004 from the Institute of Medicine discusses key aspects of nurses' working environments, including the effect of extended hours and workload on patient safety outcomes. As caregivers, nurses play an important role in patient safety and quality of care. They represent the frontline surveillance system in many healthcare settings and can detect errors before the patient can be harmed. The risks for patient infections, occupational illnesses among nursing staff, and the possible spread of infection to nurses' family members can be avoided or reduced if working conditions are supportive. Most nursing staff are women, substantial numbers of whom appear to be leaving the field for other career opportunities. Policymakers must closely monitor emerging trends in the nursing workforce to weigh possible benefits of legislation to increase the supply of nurse workers and reduce adverse patient outcomes.

## Overview of Health System Staffing Challenges

The nurse shortage is a major issue affecting the healthcare system. The demand for nursing services is expected to grow 40% by 2020, while the number of nurses is projected to grow 6%. Over the past few years, health care systems have gone through numerous reengineering and redesigning processes intended to improve the efficiency of the system. These have generally resulted in heavier workloads for nurses and have, in some instances, triggered the departure of experienced nurses. In contrast to hospitals (where downsizing and closures have been substantial over the past decade), the numbers of nursing homes and nursing home beds have substantially increased, as have the number of residents >85 years of age.

Low staffing levels increase nurses' workloads and are associated with heightened risks for adverse patient events, which may generate excess costs to the healthcare system. Healthcare settings need to use errors as opportunities for improvement, while making error detection and prevention the responsibility of all involved in the interdisciplinary collaborative work of patient care.

## Outcomes of Intensive Care Unit Conditions

An investigation supported by the Agency for Healthcare Research and Quality is studying the effects of various working conditions in intensive care units (ICUs) on elderly patient safety outcomes, healthcare worker safety, and factors related to turnover of critical-care registered nurses (RNs). The working conditions being examined include staffing levels and organizational climate, and the outcomes for the patient include healthcare-acquired infection, length of stay, death, and disposition at discharge. Healthcare worker safety includes musculoskeletal injuries, blood and body fluid exposure, sick days, and disability days. The study involved a voluntary sample of 2,330 RNs (response rate 41%) employed across the nation (east 38%, midwest 31%, west 22%, and unknown 9%) in 68 different hospitals.

Preliminary findings indicate that adverse organizational conditions are widespread and may be linked to patient safety outcomes. Overall, study results to date suggest that interventions and policies aimed at improving working conditions in ICUs may decrease turnover, improve patient safety, and reduce healthcare costs. Further research is needed to link these working conditions to patient and healthcare worker safety.

## Nurse Staffing and Adverse Patient Outcomes

The deepening nurse shortage internationally has a number of underlying causes, including a special convergence of demographic and financial forces in health care that can be likened to a "perfect storm." Recent intense public concern about healthcare safety is an added element that has made identifying the consequences of nurse staffing levels for patients particularly urgent. A growing body of literature ties lower nurse staffing levels to higher rates of death and complications of care, including healthcare-related infections, independent of patient and hospital characteristics. Methods and problems in measuring staffing are debated in the literature. Mounting evidence demonstrates that nurse staffing is a public safety issue that will require a multifaceted policy approach.

